# Supplementary material for: Is Nigeria on course to achieve universal health coverage in the context of its epidemiological and financing transition? A knowledge, capacity and policy gap analysis (a qualitative study)
Source: BMJ Open. 2023 Mar 10;13(3):e064710. doi: 10.1136/bmjopen-2022-064710 (PMC10008219; doi:10.1136/bmjopen-2022-064710)
Supplement: Supplementary data [file bmjopen-2022-064710supp001.pdf]

Supplementary file 1: Topic guide

1. What is your main role/function in this MDA, organization or institution?
2. Tell me about the 4D's (disease, demographic, donor financing, and development financing transitions) and its potential impact on the Nigerian health system?
3. From your perspective and experience, how is Nigeria faring with meeting UHC goals? around financial protection, access, benefit packages in the context of the 4Ds.
4. Describe the relevant laws, policies and programs at both national and state levels geared towards UHC?
5. From your perspective as a key stakeholder in the health sector, describe the knowledge gaps (if any) among key actors that may delay UHC advancement in Nigeria?
6. How can these knowledge gaps that you mentioned be filled/what are your recommendations for addressing these knowledge gaps you have highlighted?
7. Tell me about your role and how it fits into the UHC agenda especially within the context of the 4Ds?
8. What key challenges do you face in terms your role in UHC initiatives?
9. Tell me about your collaborations regarding UHC. Can you tell me more about the one that stood out the most for you?
10. How do you perceive interinstitutional relationships for UHC?
11. From previous collaborations with other organisations/MDAs, what would you improve or what could have been done better to maximize results
12. How has the 4Ds affected your organization/MDA's strategic plan and operations?
13. What are your perspectives on the current National Health Financing policy and strategies?
14. Describe your experience or other stakeholders' experiences with gaining access to decision makers to influence policies, implementation, or decisions?

Ogundeji YK, *et al. BMJ Open* 2023; 13:e064710. doi: 10.1136/bmjopen-2022-064710
